# Use of deep learning in forensic sex estimation of virtual pelvic models from the Han population

**DOI:** 10.1080/20961790.2021.2024369

**Published:** 2022-02-17

**Authors:** Yongjie Cao, Yonggang Ma, Xiaotong Yang, Jian Xiong, Yahui Wang, Jianhua Zhang, Zhiqiang Qin, Yijiu Chen, Duarte Nuno Vieira, Feng Chen, Ji Zhang, Ping Huang

**Affiliations:** a Shanghai Key Laboratory of Forensic Medicine, Shanghai Forensic Service Platform, Academy of Forensic Science, Ministry of Justice, Shanghai, China; b Department of Forensic Medicine, Nanjing Medical University, Nanjing, China; c Department of Medical Imaging, 3201 Hospital of Xi’an Jiaotong University Health Science Center, Hanzhong, China; d Department of Forensic Pathology, Shanxi Medical University, Taiyuan, China; e Department of Forensic Medicine, Guizhou Medical University, Guiyang, China; f Institute of Legal Medicine, Faculty of Medicine, University of Coimbra, Coimbra, Portugal

**Keywords:** Forensic sciences, forensic anthropology, sex estimation, pelvis, deep learning, convolutional neural network

## Abstract

Accurate sex estimation is crucial to determine the identity of human skeletal remains effectively. Here, we developed convolutional neural network (CNN) models for sex estimation on virtual hemi-pelvic regions, including the ventral pubis (VP), dorsal pubis (DP), greater sciatic notch (GSN), pelvic inlet (PI), ischium, and acetabulum from the Han population and compared these models with two experienced forensic anthropologists using morphological methods. A Computed Tomography (CT) dataset of 862 individuals was divided into the subgroups of training, validation, and testing, respectively. The CT-based virtual hemi-pelvises from the training and validation groups were used to calibrate sex estimation models; and then a testing dataset was used to evaluate the performance of the trained models and two human experts on the sex estimation of specific pelvic regions in terms of overall accuracy, sensitivity, specificity, F1 score, and receiver operating characteristic (ROC) curve. Except for the ischium and acetabulum, the CNN models trained with the VP, DP, GSN, and PI images achieved excellent results with all the prediction metrics over 0.9. All accuracies were superior to those of the two forensic anthropologists in the independent testing. Notably, the heatmap results confirmed that the trained CNN models were focused on traditional sexual anatomic traits for sex classification. This study demonstrates the potential of AI techniques based on the radiological dataset in sex estimation of virtual pelvic models. The excellent sex estimation performance obtained by the CNN models indicates that this method is valuable to proceed with in prospective forensic trials.

Deep learning can be a promising alternative for sex estimation based on the pelvis in forensic anthropology.

The deep learning convolutional neural network models outperformed two forensic anthropologists using classical morphological methods.

The heatmaps indicated that the most known sex-related anatomic traits contributed to correct sex determination.

## Introduction

Accurate sex estimation of skeletal remains is fundamental to individual identification in forensic anthropology, by which other biological elements (e.g. ancestry, age, and stature) could be determined [1–4]. The pelvis has always been considered as the most reliable among all human bones for its remarkable sex dimorphism, primarily affected by the functions of bipedal locomotion and parturition [3, 5–8]. Traditionally, forensic anthropologists estimate sex by empirically evaluating morphological features of the pelvis (e.g. ventral arc and subpubic contour). In some cases, however, they must deal with parts of the pelvis when corpses are poorly preserved during mass disasters or by carnivore scavenging activities [9–11]. These could make pelvis-based sex estimation more challenging. Therefore, it may be beneficial to establish additional objective methods to supplement routine morphological observation.

Current classical morphological sex estimation approaches mainly originate from American and English skeletal collections [12–14]. The individuals from these collections were born in the second half of the 18th and 19th centuries [15, 16]. Although these approaches have been widely validated, it remains questionable whether their implementation can directly apply to the Han nationality due to environmental influences on skeletal development and population-specificity [13, 15, 17–21]. Since Computed Tomography (CT) scanners have been widely used in hospitals, collecting adequate forensic anthropological reference data in a contemporary population is convenient. Besides, the CT-based three-dimensional (3D) reconstruction technique is sufficient to portray many morphological features on their actual skeletal counterparts [22, 23]. Therefore, virtual skeletal remains constructed by CT scanning could be recognised as a potential candidate for forensic anthropology and even as a substitute for traditional skeletal collection in some specific situations [24, 25].

Artificial intelligence (AI) is likely to affect many fields by accomplishing tasks considered difficult for human experts [26–29]. Powered by its advances in computation on vast amounts of datasets, deep learning has gained considerable attention for realising AI, especially in the domain of medical image recognition [30]. As exemplified by the study from Kermany et al. [29], a deep learning model with transfer-learning was used as a diagnostic tool to screen patients with common treatable blinding retinal diseases. Additionally, several studies reported the application of deep learning as a promising method in forensic anthropology. For example, Spampinato *et al.* [31] proposed several deep-learning approaches to perform automatic skeletal bone age assessment on 1 391 left-hand X-ray scans of children; the results showed an average discrepancy of about 0.8 years between manual and automated evaluation. Li *et al.* [32] used 1 875 clinical pelvic radiographs to develop a convolutional neural network (CNN) for bone age estimation; the model achieved excellent performance with the mean absolute error of 0.89 years in test samples. Notably, only a few studies have focused on applying CNN models for sex estimation, using radiographs of hands and wrists and CT reconstructions of skulls [33, 34].

Deep learning has the advantage of hierarchically extracting feature representations from the input imaging data. This study aimed to train the CNN models (GoogLeNet Inception V4) for sex estimation based on virtual hemi-pelvic bones, reconstructed with CT scanning from 862 individuals of Han nationality. Moreover, a comparative study was performed between the trained CNN models and human experts in independent testing.

## Materials and methods

### Study population and 3D model reconstruction

This work is a retrospective study based on 862 (females: 437; males: 425) pelvic CT scans retrieved from the database of the Department of Medical Imaging of Hanzhong Hospital. The scans were randomly selected from the adult Han Chinese visiting the hospital for CT-imaging of the pelvis between 2015 and 2017. These individuals aged from 20 to 85 years represented the Han population in this context. The pelvises with diseases, deformities, and injuries were eliminated, and only information of sex, age, and ancestry was retained. The study was approved by the Ethics Committees of Nanjing Medical University and Academy of Forensic Science, Ministry of Justice, and undertaken according to the Declaration of Helsinki. The committees exempted written informed consent from patients because of the anonymity of the participants’ details and the retrospective nature of this study. The permission to use the information in this database for the purposes of this research was obtained from the dataset owner, Hanzhong Hospital.

CT acquisition was performed on an Optima CT660 (GE Healthcare, Chicago, IL, USA) with the tube voltage of 120 kV, tube current of 300 mA, slice thickness of 1.25 mm, and spiral pitch factor of 0.98. The scans of the pelvises were manually reconstructed into 3D virtual skeletal models with the Mimics software (Materialise Co., Leuven, Belgium) by a single researcher using comparable standard protocols. Then the virtual ossa coxae were separated from their adjacent soft tissues and bone structures with the Hounsfield unit measurements from 226 to 3 071.

### 2D Image acquisition and preprocessing

Only the left sides of the ossa coxae were included in this study. Eighty percentage of the samples (female: 350; male: 340) as training (female: 263; male: 255) and validation datasets (female: 87; male: 85) were randomly selected for CNN model calibration while the remaining 20% ones (female: 87; male: 85) as a testing dataset were used for a comparative study between the CNN models and two human experts.

Six specific regions, including the ventral pubis (VP), dorsal pubis (DP), greater sciatic notch (GSN), pelvic inlet (PI), ischium, and acetabulum, were manually cut off from the virtual hemi-pelvic models by a single researcher. The ventral and dorsal profiles of the pubic bone, GSN and PI plane, the ischium’s ventral profile, and the acetabular rim were oriented perpendicular to the viewer. Their corresponding 2D images were manually captured and downsampled to 255 × 255 pixels. Our training and validation datasets were relatively limited for deep learning. Therefore, data augmentation, randomly changing the images’ contrast, brightness, and rotation angles (including 90°, 180°, and 270°), was performed to inflate the dataset size. This technique is a powerful and widely used method to improve a model’s generalisability over unforeseen data. In this way, the images were increased fourfold for the CNN training.

### CNN models training and validation

The GoogLeNet Inception V4 architecture [35], whose internal weights had been pre-trained on the ImageNet dataset containing 1.28 million images with 1 000 categories, was adopted for sex estimation on the virtual hemi-pelvic images using transfer learning. Transfer learning is another approach to addressing a lack of data by using the pre-trained weights as the initial weights in a new task. The CNNs were trained and assessed internally using the images from the training and validation datasets. During the training, the loss values mirroring model performance were reduced iteratively by an Adadelta algorithm [36] with a learning rate of 0.01 and a mini-batch size of 64. Learning rate is perhaps the most critical hyperparameter in deep learning, controlling how quickly the model is updated and how much the weights are changed in each training epoch. Batch size is also a hyperparameter that defines the number of images to be fed into the model at each step. A learning rate decay factor of 0.8 and a decay step of 10 implied that the learning rate decreased to 80% after 10 epochs. Heatmap analysis based on the Guided Backpropagation algorithm [37] was used to determine the pixel regions on the hemi-pelvic images contributing to sex classification by the CNN models. All the experiments were implemented on an Ubuntu 16.04 standard computer equipped with an NVIDIA Titan Xp 12 GB graphic processing unit (GPU), an Intel I7 8700 K central processing unit (CPU), and 32 GB random access memory (RAM).

### Sex estimation on virtual hemi- pelvises of the independent testing dataset by CNN models and human experts

The trained CNN model was fed with the virtual hemi-pelvic region images in the independent testing dataset, then the probability values for sex estimation were output. By artificially setting the threshold to 0.5, a female could be determined when the probability value was higher than 0.5; and a male could be distinguished when the probability value was less than 0.5. Two experienced anthropologists (A, B, with 12, 8 years of forensic anthropology experience, respectively) were given the independent images and then evaluated empirically their sexes being female or male based on previously reported methods. For VP, DP, and GSN, the images were scored on an ordinal scale from 1 to 5 (1 = hyperfeminine, 2 = feminine, 3 = intermediate, 4 = masculine, and 5 = hypermasculine) to reflect the variation in the expression of morphological traits according to the method previously published by Klales *et al.* [14] and Walker [13]. The images with scores ≤2 were determined as females, those with ≥ 4 as males, and those = 3 as undetermined individuals. As for PI, ischium, and acetabulum, the anthropologists estimated the sex on a binary scale based on Rogers and Saunders [38] and Bruzek [4]. According to Rogers and Saunders, the male PI is heart-shaped, while the female expression is elliptical; and the acetabulum in males is large and oriented laterally, whereas in females, it is said to be small and directed more anterolaterally. Bruzek [4] reported that the ischium length is longer than pubis length in males while shorter in females.

### Statistical analysis

We used the receiver operating characteristic (ROC) curve to show the classification ability of the deep learning models in sex estimation. The ROC curve was created by plotting the sensitivity against the specificity by varying the predicted probability threshold, and then the area under the ROC curve (AUC) value was achieved. The females and males were artificially prescribed as true positives and true negatives. Sensitivity was calculated as the fraction of the correctly identified females, and specificity was calculated as the fraction of the males who were correctly identified. 95%CIs for sensitivity and specificity were calculated with the Clopper–Pearson method. The DeLong nonparametric statistical test implemented in MedCalc (Version 19.2.0) was used to assess significant differences among the AUC values [39].

The intra- and interobserver agreements between the two observers were quantified with the Weighted Kappa (*k*) for the qualitative ordinal ranked data of the VP, DP, and GSN and the Cohen’s Kappa (*k*) for the binary data of the PI, ischium, and acetabulum. A score = 0.00 shows no agreement, 0.01–0.20 indicates slight agreement, 0.21–0.40 is fair, 0.41–0.60 is moderate, 0.61–0.80 is substantial, and 0.81–1.00 means almost perfect agreement. Statistical analysis was performed using SPSS 25.0 (IBM, Armonk, NY, USA).

## Results

### CNN models training and validation

**Figure 1.**
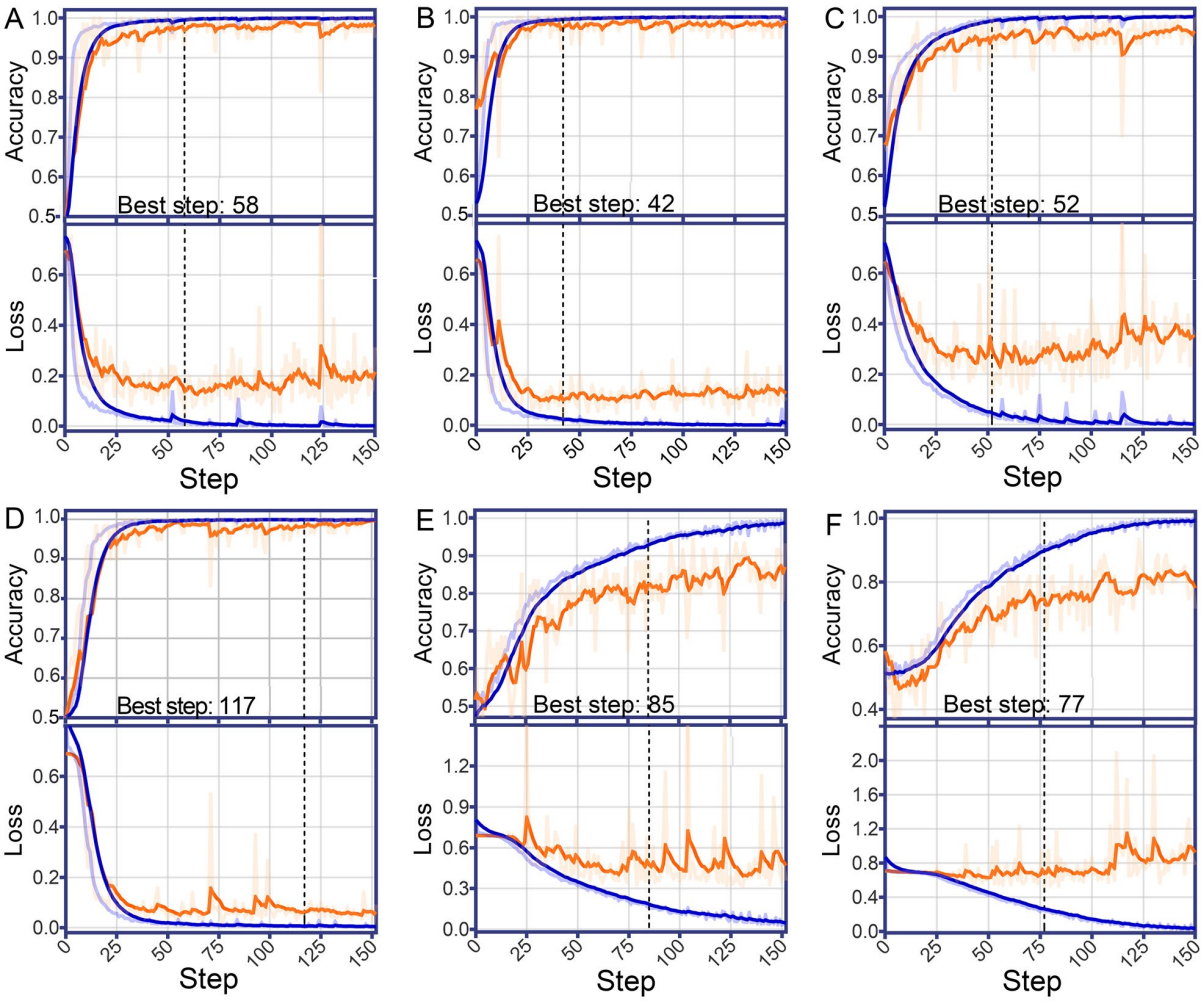
Performance of convolutional neural network models in training and validation based on specific hemi-pelvic regions, including the ventral pubis (A), dorsal pubis (B), greater sciatic notch (C), pelvic inlet (D), ischium (E) and acetabulum (F). Accuracy and loss on the training (blue curves) and validation (orange curves) datasets are plotted as functions of training steps. All the curves were smoothed with a factor of 0.8 to visualise their trends. The training was terminated early (black dotted lines) to prevent model overfitting when the relatively highest accuracy and lowest loss values were reached.

The training samples were iteratively fed into the Inception V4 to modify the model’s weights and biases in different layers. The trained model was evaluated at each step on the validation dataset regarding classification accuracy and validation loss. The loss function was a metric that distilled all aspects of the CNN model into a single number called loss value, which we sought to minimise during deep learning training. Low validation loss and high accuracy empirically implied an excellent efficacy. In Figure 1 and Table 1, the best models of VP, DP, and GSN converged at about 50 steps with a training time of about 35 min, which was shorter than the other three models. Nevertheless, the maximum training time for the six models was about 2 h. As for the internal validation, the accuracies of the six models ranged from 74.6% (acetabulum) to 98.2% (PI), with most of the CNN models having validation loss values below 0.35. Only the validation loss values of the ischium and acetabulum models was higher than 0.50, implying that their sex estimation efficacy could be unpromising.

**Table 1.** Performance of convolutional neural network models on sex estimation during training and validation.

Pelvic region	Steps	Training accuracy	Training loss	Validation accuracy	Validation loss	Time (min)
Ventral pubis	58	0.995	0.021	0.969	0.139	37.6
Dorsal pubis	42	0.993	0.025	0.972	0.100	28.6
Greater sciatic notch	52	0.988	0.050	0.947	0.348	34.8
Pelvic inlet	117	0.998	0.006	0.982	0.062	74.7
Ischium	85	0.931	0.191	0.825	0.504	131.1
Acetabulum	77	0.898	0.267	0.746	0.719	54.4

### Independent prediction on the testing samples with CNN models

When the six CNN models were fully trained using the training and validation datasets, the models’ performance was then evaluated independently with the testing dataset. As shown in Table 2, the prediction metrics demonstrated the excellent estimation performance of the CNNs, with AUC values ranging from 0.930 (95%CI 0.891–0.968) to 1.000 (95%CI 1.000–1.000) for the VP, DP, GSN, PI, and ischium, better than acetabulum (AUC: 0.822; 95%CI 0.759–0.884). The comparison of the confidence intervals of the AUC values using the DeLong test reflected that the VP, DP, and PI yielded higher AUC values than others (*P* < 0.05). No significant differences in AUC values were found among these three models (*P* > 0.05). Additionally, there was no remarkable statistical significance of AUC values between GSN and ischium models (*P* > 0.05), both of which had higher AUC values than the acetabulum model (*P* < 0.05) that presented the lowest AUC value of 0.822. The results further implied that some inherent traits in the VP, DP, and PI might be the main contributors to differentiating males from females. Table 2 also showed the accuracies, sensitivities, specificities, and F1 scores of the six CNN models in the independent testing. The sex estimation accuracy of each CNN model in the independent testing was similar with that in the internal validation procedure (VP: 100.0% *vs* 96.9%; DP: 98.8% *vs* 97.2%; GSN: 93.0% *vs* 94.7%; PI: 96.5% *vs* 98.2%; ischium: 86.0% *vs* 82.5%; acetabulum: 73.8% *vs* 74.6%).

**Table 2.** Performance of convolutional neural network models on independent sex estimation.

Pelvic region	Accuracy	Sensitivity	Specificity	F1 score	AUC
Ventral pubis	1.000 (0.979, 1.000)	1.000 (0.958, 1.000)	1.000 (0.989, 1.000)	1.000	1.000 (1.000, 1.000)
Dorsal pubis	0.988 (0.959, 1.000)	0.989 (0.938, 1.000)	0.988 (0.936, 1.000)	0.989	0.999 (0.999, 1.000)
Greater sciatic notch	0.930 (0.881, 0.963)	0.920 (0.841, 0.967)	0.941 (0.868, 0.981)	0.930	0.965 (0.936, 0.995)
Pelvic inlet	0.965 (0.926, 0.987)	0.989 (0.989, 1.000)	0.941 (0.941, 0.981)	0.967	0.998 (0.995, 1.000)
Ischium	0.860 (0.800, 0.909)	0.908 (0.827, 0.959)	0.812 (0.712, 0.888)	0.868	0.930 (0.891, 0.968)
Acetabulum	0.738 (0.666, 0.802)	0.770 (0.668, 0.854)	0.706 (0.597, 0.800)	0.749	0.822 (0.759, 0.884)

Note: All the metrics were calculated using an untuned threshold value of 0.5. The females and males were artificially prescribed as true positives and true negatives. Sensitivity and specificity were calculated as the fraction of females and males who were correctly identified in the true condition. Key descriptive statistics include total samples (*n* = 172), true female (positive) findings (*n* = 87), and true male (negative) findings (*n* = 85). The numbers in parentheses are the 95% confidence intervals. AUC: the area under the receiver operating characteristic curve.

**Figure 2.**
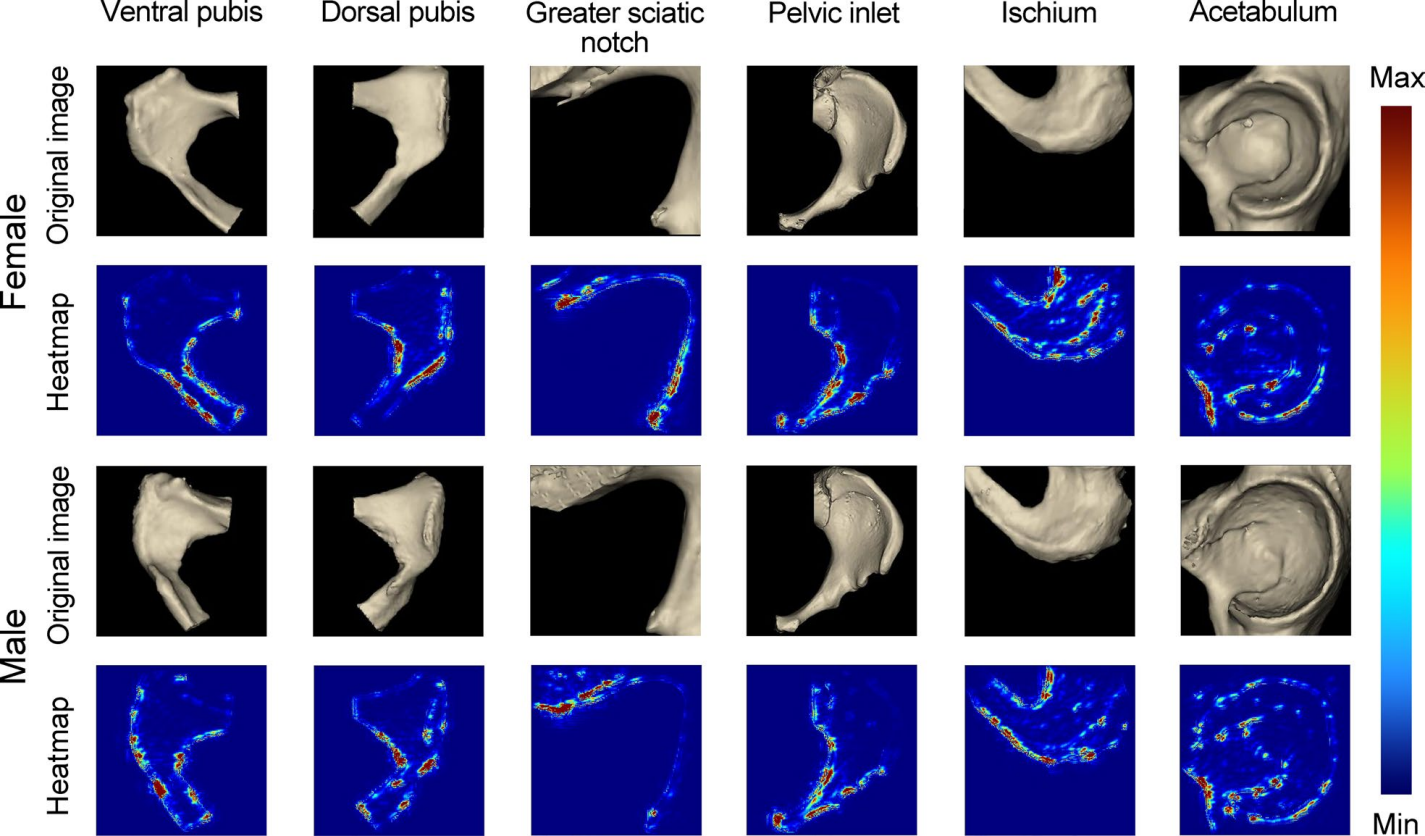
Guided Backpropagation heatmaps on the ventral pubis, dorsal pubis, greater sciatic notch, pelvic inlet, ischium, and acetabulum. Red indicates areas that contribute highly to sex estimation classification, while blue represents little contribution.

Despite excellent performance in visual classification, neural networks are also called “black boxes” that lack transparency. In Figure 2, we selected some examples of the positive outputs containing the regions of interest determined by our deep learning classifiers through the Guided Backpropagation heatmap test. In the VP and DP, the inferior margin of the pubic ramus and the whole pubis shape contributed highly to sex estimation. Based on the heatmaps from the VP, DP, ischium, and acetabulum, it was implied that the obturator foramen contributed somewhat to the pelvic sex estimation.

### Independent prediction on the testing samples with morphological evaluation by human experts

Table 3 demonstrated almost perfect levels of intraobserver agreements for VP, DP, and GSN (Anthropologist A: 0.864–0.891; Anthropologist B: 0.820–0.899), and substantial agreements for PI, ischium, and acetabulum (Anthropologist A: 0.759–0.770; Anthropologist B: 0.724–0.809). There were substantial interobserver agreements for all the traits (Anthropologist A & B: 0.651–0.815). These suggested the relative reliability of the sex estimation made by human experts. As shown in Table 4, anthropologist A and B presented similar prediction metrics, with accuracies ranging from 72.7% (65.4%–79.2%) to 80.2% (73.5%–85.9%), sensitivities ranging from 71.3% (60.6%–80.5%) to 88.5% (79.9%–94.3%), and specificities 56.5% (45.3%–67.2%) to 81.2% (71.2%–88.8%).

**Table 3.** Intra- and interobserver variability among human experts on traditional morphological sex estimation.

Pelvic region	Intraobserver error rates (left: expert A; right: expert B)		Interobserver error rates
Ventral pubis	0.888 (0.850, 0.926)	0.820 (0.771, 0869)	0.815 (0.768, 0.863)
Dorsal pubis	0.891 (0.851, 0.931)	0.824 (0.770, 0.879)	0.760 (0.702, 0.819)
Greater sciatic notch	0.864 (0.821, 0.907)	0.899 (0.863, 0.934)	0.770 (0.718, 0.822)
Pelvic inlet	0.770 (0.696, 0.844)	0.809 (0.717, 0.901)	0.672 (0.560, 0.784)
Ischium	0.759 (0.683, 0.835)	0.744 (0.638, 0.850)	0.659 (0.545, 0.773)
Acetabulum	0.760 (0.684, 0.876)	0.724 (0.624, 0.824)	0.651 (0.537, 0.765)

Note: The numbers in parentheses are the 95% confidence intervals. The intra- and interobserver agreements were quantified with the Weighted Kappa (*k*) for the qualitative ordinal ranked data of the ventral pubis, dorsal pubis, and greater sciatic notch and the Cohen’s Kappa (*k*) for the binary data of the pelvic inlet, ischium, and acetabulum.

**Table 4.** Performance of traditional morphological methods on independent sex estimation.

Pelvic region	Expert	Accuracy	Sensitivity	Specificity	F1 score
Ventral pubis	A	0.791 (0.722, 0.849)	0.862 (0.771, 0.927)	0.718 (0.610, 0.810)	0.806
	B	0.767 (0.697, 0.828)	0.885 (0.799, 0.943)	0.647 (0.536, 0.748)	0.794
Dorsal pubis	A	0.802 (0.735, 0.859)	0.862 (0.771, 0.927)	0.741 (0.635, 0.830)	0.815
	B	0.727 (0.654, 0.792)	0.885 (0.799, 0.943)	0.565 (0.453, 0.672)	0.766
Greater sciatic notch	A	0.744 (0.672, 0.808)	0.713 (0.606, 0.805)	0.776 (0.673, 0.860)	0.738
	B	0.767 (0.697, 0.828)	0.724 (0.618, 0.815)	0.812 (0.712, 0.888)	0.759
Pelvic inlet	A	0.779 (0.710, 0.839)	0.828 (0.732, 0.900)	0.729 (0.622, 0.820)	0.791
	B	0.791 (0.722, 0.849)	0.828 (0.732, 0.900)	0.753 (0.647, 0.840)	0.800
Ischium	A	0.773 (0.703, 0.834)	0.816 (0.719, 0.891)	0.729 (0.622, 0.820)	0.785
	B	0.756 (0.685, 0.818)	0.805 (0.706, 0.882)	0.706 (0.590, 0.800)	0.769
Acetabulum	A	0.767 (0.697, 0.828)	0.759 (0.655, 0.844)	0.776 (0.673, 0.860)	0.767
	B	0.756 (0.685, 0.818)	0.793 (0.693, 0.873)	0.718 (0.610, 0.810)	0.767

Note: The females and males were artificially prescribed as true positives and true negatives. Sensitivity and specificity were calculated as the fraction of females and males who were correctly identified in the true condition. Key descriptive statistics include total samples (*n* = 172), true female (positive) findings (*n* = 87), and true male (negative) findings (*n* = 85). The numbers in parentheses are the 95% confidence intervals.

### Comparison of sex estimation on the testing samples between CNNs and human experts

**Figure 3.**
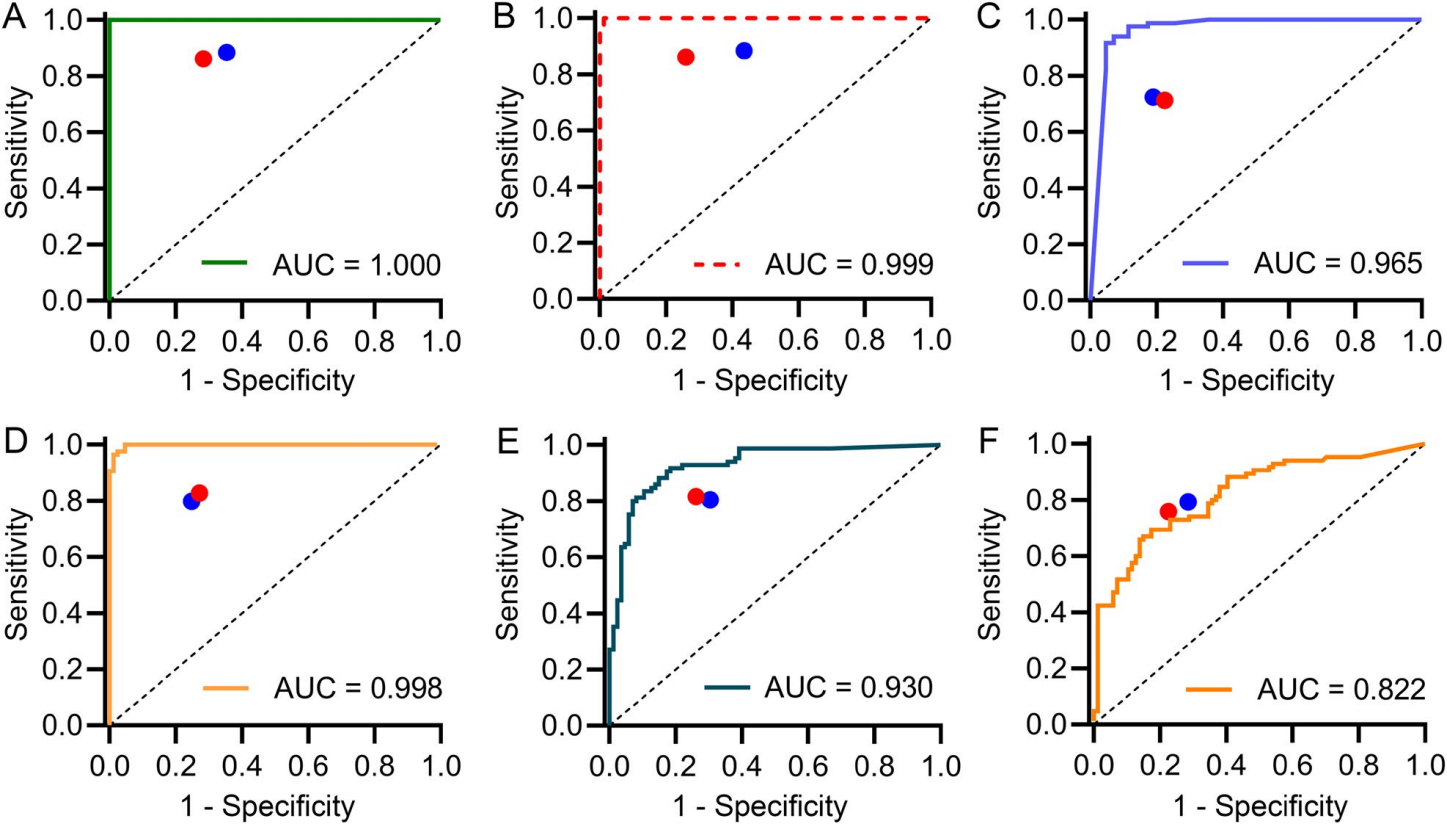
Performance comparison between convolutional neural network models and human experts (red dot: human expert A; blue dot: human expert B) for sex estimation based on the ventral pubis (A), dorsal pubis (B), greater sciatic notch (C), pelvic inlet (D), ischium (E) and acetabulum (F). Except for the acetabulum, most models achieve superior performance to two anthropologists as their specificity–sensitivity points lay below the corresponding model’s receiver operating characteristic curve. AUC: the area under the receiver operating characteristic curve.

As shown in Figure 3, the points (1-specificity, sensitivity) of both human experts, except for the acetabulum, laid below the ROC curves of their corresponding CNN models, implying that the CNN models achieved superior overall performance in sex estimation than human experts in the five selected pelvic regions other than the acetabulum. As for the acetabulum, the CNN model and the anthropologists had almost comparable sensitivity and specificity. Except for the acetabulum, the CNN models performed better than the experts in terms of accuracy, sensitivity, specificity, and F1 scores (Tables 2 and 4).

## Discussion

In some cases, forensic anthropologists have no access to regular and entire pelvis bones, which could be partially destroyed, missed, or deformed due to antemortem or postmortem injury during wars, air, or traffic crashes, dismember, and animal gnawing. It is challenging to make accurate sex estimation in the above scenarios, regardless of using morphological or metric methods. Besides, some sex estimation methods based on ancient skeletal collection may not be applicable to other current populations due to significant variations of ancestry, environment, and nutrition [12–14, 17, 18, 40]. These all would prevent forensic anthropologists from making effective sex judgments, thereby misleading further construction of skeletal biological profiles and police investigation. To deal with these situations, we investigated the performance of the CNN models on the specific regions of 3D virtual hemi-pelvis samples reconstructed from the contemporary Han CT scans, including the VP, DP, GSN, PI, ischium, and acetabulum. We then compared the performance of these models to that of two experienced forensic anthropologists using traditional methods.

The findings of this retrospective study showed that most CNN models tested in the independent hemi-pelvic dataset could achieve high accuracies, sensitivities, and specificities in sex estimation, which were comparable to those in the internal validation procedure. This demonstrated the generalisability of our deep learning models to the unforeseen samples without significant performance differences of overfitting. In the case of overfitting, the deep learning model is so well-trained on the training data that it cannot adapt to new data. In contrast, an underfitted model fails to make accurate predictions even on the training data. In this context, our acetabulum model should be considered as underfitted according to its AUC value and accuracy in the testing dataset. We do not know whether this model’s underfitting is mainly related to the limited data size or to the sexually dimorphic nature of the acetabulum, which should be clarified in future investigations.

The results also showed that most CNN models outperformed human experts on sex estimation except acetabulum in terms of accuracy, sensitivity, and specificity. In addition, CNN models could be well-trained in a few hours, whereas a qualified anthropologist may require years of professional training. The heatmap based on the Guided Backpropagation algorithm demonstrated that these models mainly concentrated on the anatomic structures that are well-known to contribute significantly to sexual classification. Indeed, several morphological studies have confirmed the validity and reliability of some traits (e.g. ventral arc, subpubic angle, pubis body shape, and subpubic contour) on the VP and DP [12, 14, 17, 19, 20, 41–45]. However, our results yielded higher classification accuracies of both pelvic regions than these studies. Moreover, it was found that the pelvis inlet has sexual dimorphism, reaching a high accuracy of 96.5%–100%, even though this anatomic trait is not widely recognised as an ideal alternative for sex estimation [46]. Compared to similar studies on the GSN [4, 13, 47], higher accuracy we obtained could be explained by the ability of the CNN models to ignore useless details such as developmental variation of marginal structures like the ischial spine and piriform tubercle, which usually makes the traditional morphological and metric method more challenging. Contrary to several other studies (accuracy ranging from 82.5% to 96.4%) [48–53], the model based on the acetabulum even cannot reach the recommended 75% accuracy boundary [54, 55], which may be highly associated with the difficulty to separate acetabulum from the femoral head during CT construction [56].

These results demonstrate not only the power of AI technology for sex estimation but also the advantages of radiological techniques on rapid data renewal, especially for contemporary populations within specific regions. Despite the high performance of our sex estimations, there are still several limitations of the proposed method. Firstly, the dataset scale is relatively small. Data augmentation and transfer learning techniques were applied in this study to overcome the challenge of lacking training data; and the sex estimation performance of the models on the independent dataset demonstrated that these techniques appeared to address the problem of sample insufficiency. In addition, several studies reported the feasibility of transfer learning to train models with limited data. Kermany *et al.* [29] found that the classifier trained with 1 000 samples using transfer learning retained comparable performance to that trained with 10 000 samples from scratch. However, Kermany *et al.* also acknowledged that the model’s performance using transfer learning would be inferior to that of a model trained on an extremely large dataset. Secondly, the samples we collected originated from one platform and primarily are north-western Han Chinese. Although most of our models exhibited excellent generalisability in the independent dataset, the applicability of our method needs to be further evaluated using the CT dataset that would be generated by various instruments, software, and population statistics and the images not derived from CT scans, such as pictures from cameras or smartphones. Future multicentre studies should include more data and expand the sets to real-world data from other resources to increase the performance and generalisability of our AI sex estimation system.

Although our computational analyses may play a role in the hemi-pelvic sex estimation, we still believe that AI will not replace the forensic anthropologists who use morphological methods, given its limitations. Firstly, for their nature of lacking interpretability and transparency, the inspiring and promising deep learning techniques should be applied with caution. The heatmap test showed that our models mainly focused on the skeletal structures with sex dimorphism; it is still impossible to explain how and why the models produce output for a particular image. Secondly, another factor limiting deep learning is the error for landmark recognition and size between virtual and dry skeletons generated during the 3D reconstruction. Some sex estimation traits, such as pre-auricular sulcus [23] and pubic symphysis scarring, may be invisible or poorly defined in the 3D virtual models. Therefore, AI techniques can be combined with manual methods to augment the capabilities of forensic anthropologists, but they cannot replace forensic anthropologists.

## Conclusion

Herein, we demonstrate the potential of AI techniques based on the radiological dataset in sex estimation of virtual hemi-pelvic models. Despite the limited number of samples available, most of the CNN models, trained with images of various hemi-pelvic anatomical regions using transfer learning, can achieve high accuracies, sensitivities, specificities, and AUC values in sex estimation, even better than human experts with significant anthropological experience. This current AI technology cannot replace forensic anthropologists, but we believe that deep learning will soon become a complementary tool for more accurate sex estimation. We would proceed with this method and test its applicability in prospective forensic trials with much large and multicentre training datasets and expand our algorithm to other types of skeletal remains with sexual dimorphism (cranium, mandible, humerus, and femur).

## References

[1] Christensen AM, Passalacqua NV. Sex estimation. In: Christensen AM, Passalacqua NV, editors. A laboratory manual for forensic anthropology. Amsterdam (The Netherlands): Elsevier Academic Press; 2018. p. 113–126.

[2] Klales AR. Practitioner preferences for sex estimation from human skeletal remains. In: Klales AR, editor. Sex estimation of the human skeleton. Amsterdam (The Netherlands): Elsevier Academic Press; 2020. p. 11–23.

[3] Harrison D. Background in adult sexual dimorphism. In: Harrison D, editor. Investigations in sex estimation. Amsterdam (The Netherlands): Elsevier Academic Press; 2019. p. 11–33.

[4] Bruzek J. A method for visual determination of sex, using the human hip bone. Am J Phys Anthropol. 2002;117:157–168. 11815949 10.1002/ajpa.10012

[5] Leutenegger W. Functional aspects of pelvic morphology in simian primates. J Hum Evol. 1974;3:207–222.

[6] Rosenberg K, Trevathan W. Birth, obstetrics and human evolution. BJOG. 2002;109:1199–1206. 12452455 10.1046/j.1471-0528.2002.00010.x

[7] Correia H, Balseiro S, De Areia M. Sexual dimorphism in the human pelvis: testing a new hypothesis. Homo. 2005;56:153–160. 16130838 10.1016/j.jchb.2005.05.003

[8] Lewin R. Human evolution: an illustrated introduction. Malden (MA): Blackwell Publishing; 2004.

[9] Moraitis K, Spiliopoulou C. Forensic implications of carnivore scavenging on human remains recovered from outdoor locations in Greece. J Forensic Leg Med. 2010;17:298–303. 20650416 10.1016/j.jflm.2010.04.008

[10] de Boer HH, Blau S, Delabarde T, et al. The role of forensic anthropology in disaster victim identification (DVI): recent developments and future prospects. Forensic Sci Res. 2019;4:303–315. 32002489 10.1080/20961790.2018.1480460 PMC6968550

[11] de Boer HH, Roberts J, Delabarde T, et al. Disaster victim identification operations with fragmented, burnt, or commingled remains: experience-based recommendations. Forensic Sci Res. 2020;5:191–201. 33224550 10.1080/20961790.2020.1751385 PMC7654639

[12] Phenice TW. A newly developed visual method of sexing the os pubis. Am J Phys Anthropol. 1969;30:297–301. 5772048 10.1002/ajpa.1330300214

[13] Walker PL. Greater sciatic notch morphology: sex, age, and population differences. Am J Phys Anthropol. 2005;127:385–391. 15693026 10.1002/ajpa.10422

[14] Klales AR, Ousley SD, Vollner JM. A revised method of sexing the human innominate using Phenice’s nonmetric traits and statistical methods. Am J Phys Anthropol. 2012;149:104–114. 22714398 10.1002/ajpa.22102

[15] Walker PL. Sexing skulls using discriminant function analysis of visually assessed traits. Am J Phys Anthropol. 2008;136:39–50. 18324631 10.1002/ajpa.20776

[16] Harrison D. Skeletal collections. In: Harrison D, editor. Investigations in sex estimation. Amsterdam (The Netherlands): Elsevier Academic Press; 2019. p. 51–69.

[17] Ubelaker D, Volk C. A test of the Phenice method for the estimation of sex. J Forensic Sci. 2002;47:19–24. 12064650

[18] Bytheway JA, Ross AH. A geometric morphometric approach to sex determination of the human adult os coxa. J Forensic Sci. 2010;55:859–864. 20384930 10.1111/j.1556-4029.2010.01374.x

[19] Klales AR, Cole SJ. Improving nonmetric sex classification for Hispanic individuals. J Forensic Sci. 2017;62:975–980. 28070893 10.1111/1556-4029.13391

[20] Gómez-Valdés JA, Menéndez Garmendia A, García-Barzola L, et al. Recalibration of the Klales et al. (2012) method of sexing the human innominate for Mexican populations. Am J Phys Anthropol. 2017;162:600–604. 28117482 10.1002/ajpa.23157

[21] Kenyhercz MW, Klales AR, Stull KE, et al. Worldwide population variation in pelvic sexual dimorphism: a validation and recalibration of the Klales et al. method. Forensic Sci Int. 2017;277:259.e1–259.e8. 28666560 10.1016/j.forsciint.2017.05.001

[22] Colman KL, Dobbe JGG, Stull KE, et al. The geometrical precision of virtual bone models derived from clinical computed tomography data for forensic anthropology. Int J Legal Med. 2017;131:1155–1163. 28185072 10.1007/s00414-017-1548-z PMC5491564

[23] Colman KL, van der Merwe AE, Stull KE, et al. The accuracy of 3D virtual bone models of the pelvis for morphological sex estimation. Int J Legal Med. 2019;133:1853–1860. 30680527 10.1007/s00414-019-02002-7 PMC6811666

[24] Thali MJ, Yen K, Schweitzer W, et al. Virtopsy, a new imaging horizon in forensic pathology: virtual autopsy by postmortem multislice computed tomography (MSCT) and magnetic resonance imaging (MRI)—a feasibility study. J Forensic Sci. 2003;48:386–403. 12665000

[25] Colman KL, Janssen MCL, Stull KE, et al. Dutch population specific sex estimation formulae using the proximal femur. Forensic Sci Int. 2018;286:268.e1–268.e8. 29548547 10.1016/j.forsciint.2017.12.029

[26] Coudray N, Ocampo PS, Sakellaropoulos T, et al. Classification and mutation prediction from non-small cell lung cancer histopathology images using deep learning. Nat Med. 2018;24:1559–1567. 30224757 10.1038/s41591-018-0177-5 PMC9847512

[27] Dunnmon JA, Yi D, Langlotz CP, et al. Assessment of convolutional neural networks for automated classification of chest radiographs. Radiology. 2019;290:537–544. 30422093 10.1148/radiol.2018181422 PMC6358056

[28] Esteva A, Kuprel B, Novoa RA, et al. Dermatologist-level classification of skin cancer with deep neural networks. Nature. 2017;542:115–118. 28117445 10.1038/nature21056 PMC8382232

[29] Kermany DS, Goldbaum M, Cai W, et al. Identifying medical diagnoses and treatable diseases by image-based deep learning. Cell. 2018;172:1122–1131.e9. 29474911 10.1016/j.cell.2018.02.010

[30] Russakovsky O, Deng J, Su H, et al. ImageNet large scale visual recognition challenge. Int J Comput Vis. 2015;115:211–252.

[31] Spampinato C, Palazzo S, Giordano D, et al. Deep learning for automated skeletal bone age assessment in X-ray images. Med Image Anal. 2017;36:41–51. 27816861 10.1016/j.media.2016.10.010

[32] Li Y, Huang Z, Dong X, et al. Forensic age estimation for pelvic X-ray images using deep learning. Eur Radiol. 2019;29:2322–2329. 30402703 10.1007/s00330-018-5791-6

[33] Yune S, Lee H, Kim M, et al. Beyond human perception: sexual dimorphism in hand and wrist radiographs is discernible by a deep learning model. J Digit Imaging. 2019;32:665–671. 30478479 10.1007/s10278-018-0148-x PMC6646498

[34] Bewes J, Low A, Morphett A, et al. Artificial intelligence for sex determination of skeletal remains: application of a deep learning artificial neural network to human skulls. J Forensic Leg Med. 2019;62:40–43. 30639854 10.1016/j.jflm.2019.01.004

[35] Szegedy C, Ioffe S, Vanhoucke V. Inception-V4, Inception-ResNet and the impact of residual connections on learning. Thirty-First AAAI Conference on Artificial Intelligence, 2017 Feb. 04–09; San Francisco, CA, USA.

[36] Zeiler MD. Adadelta: an adaptive learning rate method. ArXiv preprint ArXiv:1212.5701. 2012.

[37] Springenberg JT, Dosovitskiy A, Brox T, et al. Striving for simplicity: the all convolutional net. ArXiv preprint ArXiv:1412.6806. 2014.

[38] Rogers T, Saunders S. Accuracy of sex determination using morphological traits of the human pelvis. J Forensic Sci. 1994;39:1047–1056. 8064263

[39] DeLong ER, DeLong DM, Clarke-Pearson DL. Comparing the areas under two or more correlated receiver operating characteristic curves: a nonparametric approach. Biometrics. 1988;44:837–845. 3203132

[40] Gonzalez PN, Bernal V, Perez SI. Geometric morphometric approach to sex estimation of human pelvis. Forensic Sci Int. 2009;189:68–74. 19442464 10.1016/j.forsciint.2009.04.012

[41] Sutherland L, Suchey J. Use of the ventral arc in pubic sex determination. J Forensic Sci. 1991;36:501–511. 2066725

[42] Lovell NC. Test of Phenice’s technique for determining sex from the os pubis. Am J Phys Anthropol. 1989;79:117–120. 2750876 10.1002/ajpa.1330790112

[43] Kelley MA. Phenice’s visual sexing technique for the os pubis: a critique. Am J Phys Anthropol. 1978;48:121–122. 623228 10.1002/ajpa.1330480118

[44] McFadden C, Oxenham MF. Revisiting the phenice technique sex classification results reported by MacLaughlin and Bruce (1990). Am J Phys Anthropol. 2016;159:182–183. 26767498 10.1002/ajpa.22839

[45] Lesciotto KM, Doershuk LJ. Accuracy and reliability of the Klales et al. (2012) morphoscopic pelvic sexing method. J Forensic Sci. 2018;63:214–220. 28382657 10.1111/1556-4029.13501

[46] Klales AR. Sex estimation using pelvis morphology. In: Klales AR, editor. Sex estimation of the human skeleton. Amsterdam (The Netherlands): Elsevier Academic Press; 2020. p. 75–93.

[47] Gómez-Valdés JA, Quinto-Sánchez M, Menéndez Garmendia A, et al. Comparison of methods to determine sex by evaluating the greater sciatic notch: visual, angular and geometric morphometrics. Forensic Sci Int. 2012;221:156.e1–156.e7. 22607977 10.1016/j.forsciint.2012.04.027

[48] Steyn M, İşcan MY. Metric sex determination from the pelvis in modern Greeks. Forensic Sci Int. 2008;179:86.e1–86.e6. 18554832 10.1016/j.forsciint.2008.04.022

[49] Papaloucas C, Fiska A, Demetriou T. Sexual dimorphism of the hip joint in Greeks. Forensic Sci Int. 2008;179:83.e1–83.e3. 18455335 10.1016/j.forsciint.2008.03.007

[50] Steyn M, Patriquin ML. Osteometric sex determination from the pelvis—does population specificity matter? Forensic Sci Int. 2009;191:113.e1–113.e5. 19665855 10.1016/j.forsciint.2009.07.009

[51] Macaluso PJ, Jr. Sex determination from the acetabulum: test of a possible non-population-specific discriminant function equation. J Forensic Leg Med. 2010;17:348–351. 20650427 10.1016/j.jflm.2010.04.011

[52] Macaluso PJ, Jr. Sex discrimination from the acetabulum in a twentieth-century skeletal sample from France using digital photogrammetry. Homo. 2011;62:44–55. 21106196 10.1016/j.jchb.2010.11.001

[53] Benazzi S, Maestri C, Parisini S, et al. Sex assessment from the acetabular rim by means of image analysis. Forensic Sci Int. 2008;180:58.e1–58.e3. 18692971 10.1016/j.forsciint.2008.06.007

[54] De Vito C, Saunders S. A discriminant function analysis of deciduous teeth to determine sex. J Forensic Sci. 1990;35:845–858. 2391476

[55] Sutter RC. Nonmetric subadult skeletal sexing traits: I. A blind test of the accuracy of eight previously proposed methods using prehistoric known-sex mummies from Northern Chile. J Forensic Sci. 2003;48:927–935. 14535657

[56] Colman KL, de Boer HH, Dobbe JGG, et al. Virtual forensic anthropology: the accuracy of osteometric analysis of 3D bone models derived from clinical computed tomography (CT) scans. Forensic Sci Int. 2019;304:109963. 31610335 10.1016/j.forsciint.2019.109963

